# Temozolomide combined with irinotecan caused regression in an adult pleomorphic rhabdomyosarcoma patient-derived orthotopic xenograft (PDOX) nude-mouse model

**DOI:** 10.18632/oncotarget.16548

**Published:** 2017-03-24

**Authors:** Kentaro Igarashi, Kei Kawaguchi, Tasuku Kiyuna, Takashi Murakami, Shinji Miwa, Scott D. Nelson, Sarah M. Dry, Yunfeng Li, Arun S. Singh, Hiroaki Kimura, Katsuhiro Hayashi, Norio Yamamoto, Hiroyuki Tsuchiya, Fritz C. Eilber, Robert M. Hoffman

**Affiliations:** ^1^ AntiCancer, Inc., San Diego, CA, USA; ^2^ Department of Surgery, University of California, San Diego, CA, USA; ^3^ Department of Orthopedic Surgery, Kanazawa University, Kanazawa, Japan; ^4^ Department of Pathology, University of California, Los Angeles, CA, USA; ^5^ Division of Hematology-Oncology, University of California, Los Angeles, CA, USA; ^6^ Division of Surgical Oncology, University of California, Los Angeles, CA, USA

**Keywords:** rhabdomosarcoma, nude mice, patient-derived orthotopic xenograft (PDOX), temozolomide, irinotecan, combination

## Abstract

Adult pleomorphic rhabdomyosarcoma (RMS) is a rare and recalcitrant, highly-malignant mesenchymal tumor in need of improved therapeutic strategies. Our laboratory pioneered the patient-derived orthotopic xenograft (PDOX) nude mouse model with the technique of surgical orthotopic implantation (SOI). We previously described the development of a PDOX model of adult pleomorphic RMS where the tumor behaved similar to the patient donor. A high-grade pleomorphic rhabdomyosarcoma from a striated muscle was previously grown orthotopically in the right biceps-femoris muscle of nude mice to establish the PDOX model. In the present study, the PDOX models were randomized into the following treatment groups when tumor volume reached 100 mm^3^: G1, control without treatment; G2, cyclophosphamide (CPA) 140 mg/kg, intraperitoneal (i.p.) injection, weekly, for 3 weeks; G3, temozolomide (TEM), 25 mg/kg, per oral (p.o.), daily, for 21 days; G4, temozolomide (TEM) 25 mg/kg, p.o., daily, for 21 days combined with irinotecan (IRN), 4 mg/kg, i.p., daily for 21 days. After 3 weeks, treatment of PDOX with TEM combined with IRN was so powerful that it resulted in tumor regression and the smallest tumor volume compared to other groups. The RMS PDOX model should be of use to design the treatment program for the patient and for drug discovery and evaluation for this recalcitrant tumor type.

## INTRODUCTION

Rhabdomyosarcoma (RMS) originates in striated muscle cells. The majority of RMS cases occur below the age of 18. Approximately 40% of soft tissue sarcomas (STS) are RMS. RMS can occur in any site on the body but is primarily found in the head, neck, orbit, genitourinary tract, genitals, and extremities [1–3]. RMS is divided into three histological subsets:

Embryonal rhabdomyosarcoma (ERMS) is the most common with approximately 60-70% of childhood cases. ERMS usually occurs in patients 4 years old or younger with 4 cases per 1 million children. Head and neck as well as the genitourinary track are most common sites. ERMS usually has morphology similar to developing muscle cells of a 6-8 week-old embryo, hence the name. ERMS also has two subtypes, botryoid and spindle cell [4, 5].

Alveolar rhabdomyosarcoma (ARMS) has an incidence of 1 case per 1 million in patients aged 0 to 19. ARMS occurs most commonly in extremities, trunk, and peritoneum and is more aggressive than ERMS. ARMS is the most common form of RMS observed in young adults and teenagers. ARMS has densely-packed, round cells that are similar to pulmonary alveoli, hence the name [5, 6].

Pleiomorphic rhabdomyhosarcoma (PRMS) comprises poorly differentiated anaplastic cells. PRMS is the most aggressive type of RMS [7]. PRMS occurs most often in adults and rarely in children. RMS is usually in the extremities [8, 9].

We previously developed a patient-derived orthotopic xenograft (PDOX) mouse model of PRMS. We compared the PDOX model to a subcutaneous (s.c.)-transplant model. An RMS from a striated muscle of a male patient was grown orthotopically in the right biceps- femoris muscle or right quadriceps muscle of nude mice to establish a PDOX model. The RMS was also grown subcutaneously in nude mice. PDOX tumors grew at a statistically-significant faster rate compared to the s.c. tumors. Recurrence after surgical resection occurred only in PDOX tumors, not in the s.c. model. Histologically, only the PDOX model was shown to be invasive. These results indicated that the PDOX model of adult RMS is malignant and the subcutaneous model is benign [10].

In the present study, we used the adult PRMS PDOX model to identify an effective drug or combination for this recalcitrant disease.

## RESULTS AND DISCUSSION

### Efficacy of cyclophosphamide (CPA), temozolomide (TEM) and TEM combined with irinotecan (IRN) on the PRMS PDOX

**Figure 1:**
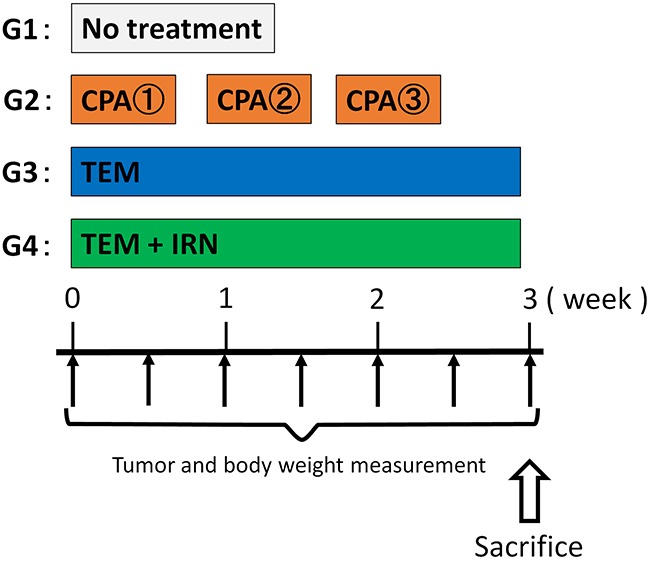
Treatment schema

**Figure 2:**
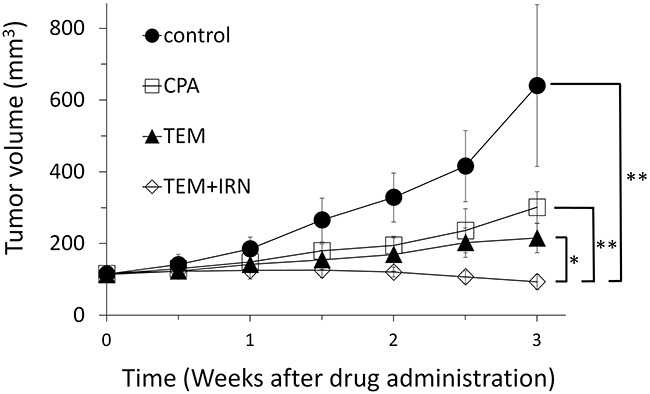
Efficacy of cyclophosphamide (CPA), temozolomide (TEM) and TEM combined with irinotecan (IRN) CPA (140 mg/kg, i.p., qw×3); TEM (25 mg/kg, p.o., qd×21); TEM (25 mg/kg, p.o., qd×21) combined with IRN (4 mg/kg, i.p., qd×21). Tumor volume was measured at the indicated time points after the onset of treatment. Please see the Materials and Methods for details. N = 8 mice/group. *P=0.0002; **P<0.0001.

**Figure 3:**
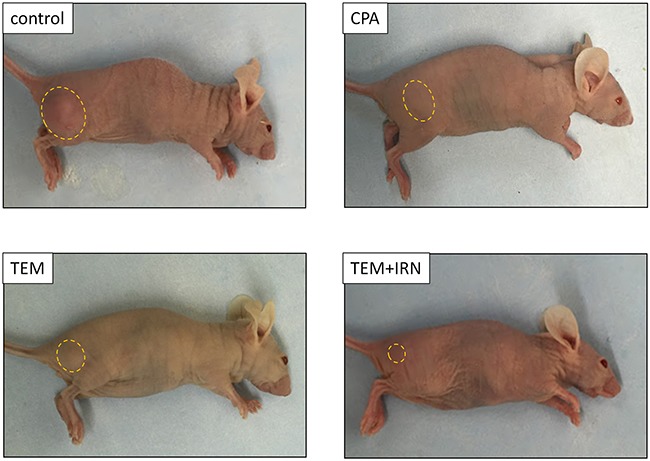
Efficacy of CPA, TEM and TEM combined with IRN Photographs of representative PDOX mouse models from each treatment group at day 21.

**Figure 4:**
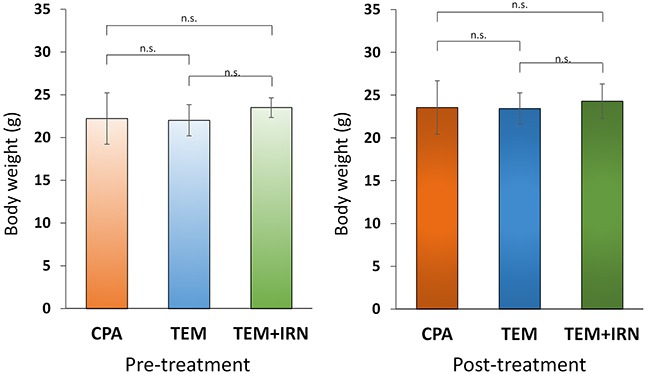
Effect of treatment on body weight Bar graph shows body weight in each group at pre-treatment and 3 weeks after drug administration.

Please see Figure 1 for treatment schema. All treatments significantly inhibited the PRMS PDOX growth compared to untreated controls on day 21 after initiation. Tumor volumes at day 21 were: control (G1): 640.6 ± 225.5 mm^3^; CPA (G2): 301.1 ± 43.6 mm^3^, *p*=0.002; TEM (G3): 215.2 ± 40.6 mm^3^, *p*=0.0004; TEM combined with IRN (G4): 93.6 ± 8.7 mm^3^, *p*<0.0001. TEM combined with IRN showed significantly more efficacy compared to other therapies evaluated: CPA (*p*<0.0001), TEM (*p*=0.0002) (Figures 2 and 3). There were no animal deaths in any group. The body weight of treated mice was not significantly different in any group (Figure 4). No significant other side effects were observed either.

### Histology of the PRMS PDOX and patient tumor

**Figure 5:**
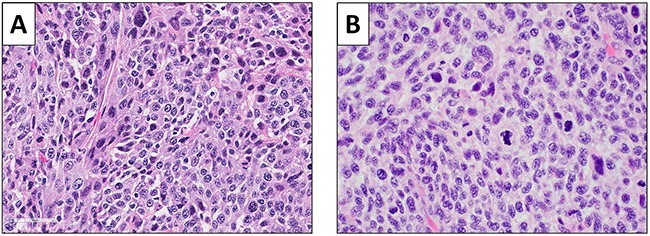
Histology of the original patient tumor and the untreated control PDOX tumor **(A)** Original patient tumor. **(B)** Untreated control PDOX tumor. See Materials and Methods for details.

A high-power photomicrograph of the original patient tumor displayed solid sheets of tumor cells characterized by pleomorphic, hyperchromatic, enlarged nuclei with coarse chromatin and moderate amounts of lightly eosinophilic cytoplasm. Numerous mitotic figures, including atypical forms are present. A high-power image of the orthotopically-implanted tumors had very similar features, including pleomorphic, hyperchromatic, enlarged nuclei with coarse chromatin and moderate amounts of lightly eosinophilic cytoplasm. Numerous mitotic figures, including atypical forms are also present (Figure 5), demonstrating the fidelity of the PDOX tumor.

The donor patient was newly diagnosed with PRMS which is considered a high-grade sarcoma. The patient had not received any prior systemic treatment. There is no consensus as to what the standard of care therapy is for this disease [11–13]. Therefore, it was decided to choose some systemic therapies that are used for RMS such as IRN and CPA. It was decided to try IRN + TMZ since this is a combination regimen that has been used for various malignancies and has shown efficacy against a wide range of tumors and several forms of sarcoma. Although this combination is not commonly used for PRMS, our unexpected results show tumor regression with this combination. This promising result makes the combination of TEM and IRN a candidate therapy for the donor patient of the PRMS PDOX, since tumor regression in the model suggested efficacy in the clinic [14]. A future study will determine if the combination of TEM and IRN is synergistic or additive in this model of PRMS.

Previously-developed concepts and strategies of highly selective tumor targeting can take advantage of molecular targeting of tumors, including tissue-selective therapy which focuses on unique differences between normal and tumor tissues [15–20].

## CONCLUSIONS

RMS is a recalcitrant disease. It usually occurs in children and young adults. In the present case, it occurred in 68-year-old man. We previously established a PDOX model of pleomorphic RMS (PRMS) [10] that is highly malignant compared to the same tumor grown subcutaneously. Therefore, we assume the PDOX model represents the patient since PRMS is a highly-malignant tumor. The present results show that the combination of TEM and IRN was so powerful that it was able to regress the RMS PDOX, indicating potential of a similar response in the patient [14].

The combination of vincristine, actinomycin D, cyclophosphamide (VAC) is sometimes used to treat RMS and will be used for future experiments comparing VAC with TEM+IRN [21]. The results of the present study suggests that potential powerful therapy can be identified for many adult PRMS patients. The PRMS PDOX can be used for discovery and evaluation of novel therapeutics for this recalcitrant disease as well.

## MATERIALS AND METHODS

### Animal care

Athymic nu/nu nude mice (AntiCancer Inc., San Diego, CA), 4–6 weeks old, were used in this study. Animals were housed in a barrier facility on a high efficiency particulate arrestance (HEPA)-filtered rack under standard conditions of 12-hour light/dark cycles. The animals were fed an autoclaved laboratory rodent diet. All animal studies were conducted with an AntiCancer Institutional Animal Care and Use Committee (IACUC)-protocol specifically approved for this study and in accordance with the principals and procedures outlined in the National Institute of Health Guide for the Care and Use of Animals under Assurance Number A3873-1. In order to minimize any suffering of the animals, anesthesia and analgesics were used for all surgical experiments. Animals were anesthetized by subcutaneous injection of a 0.02 ml solution of 20 mg/kg ketamine, 15.2 mg/kg xylazine, and 0.48 mg/kg acepromazine maleate.

### Patient-derived tumor

A 68-year-old male diagnosed with pleomorphic RMS had a large primary tumor in the right high thigh previously underwent surgical resection at Department of Surgery, University of California, Los Angeles (UCLA). The patient did not receive any chemotherapy or radiotherapy prior to surgery. Written informed consent was obtained from the patient as part of a UCLA Institutional Review Board (IRB #10-001857)-approved protocol [10].

### Surgical orthotopic implantation (SOI) to establish an RMS PDOX model

Our laboratory pioneered the PDOX nude mouse model with the technique of surgical orthotopic implantation (SOI), including pancreatic [22–25], breast [26], ovarian [27], lung [28], cervical [29], colon [30–32], stomach [33], sarcoma [34–38], and melanoma [39–41].

The PRMS tumor was previously established at AntiCancer, Inc. [10]. Tumors were initially grown subcutaneously after transplantation of 5 mm fragments. After 3 weeks growth, tumors were harvested and cut into small fragments (3-4 mm). After nude mice were anesthetized, a 5 mm skin incision was made on the right high thigh, then the biceps femoris or quadriceps was split to make space for the tumor. A single tumor fragment was implanted orthotopically into the space to establish a PDOX model [10]. The wound was closed with 6-0 nylon suture (Ethilon, Ethicon, Inc., NJ, USA).

### Treatment design

PRMS PDOX mouse models were randomized into 4 groups of 8 mice each: G1, control without treatment; G2, CPA 140 mg/kg, i.p., qw×3; G3, TEM 25 mg/kg, p.o., qd×21; G4, TEM 25 mg/kg, p.o., qd×21 combined with IRN 4 mg/kg, i.p., qd×21. Tumor length, width and mouse body weight were measured twice in a week. Tumor volume was calculated by following formula: Tumor volume (mm^3^) = length (mm) × width (mm) × width (mm) × 1/2. Data are presented as mean ± SD.

### Histological analysis

Fresh tumor specimens were fixed in 10% formalin and embedded in paraffin before sectioning and staining. Tissue sections (3 μm) were deparaffinized in xylene and rehydrated in an ethanol series. Hematoxylin and eosin (H&E) staining was performed according to standard protocol. Histological examination was performed with a BHS system microscope. Images were acquired with INFINITY ANALYZE software (Lumenera Corporation, Ottawa, Canada).
